# Effects of ultra‐high‐temperature processes on metabolite changes in milk

**DOI:** 10.1002/fsn3.3350

**Published:** 2023-04-06

**Authors:** Ge Bai, Long Cheng, Liying Peng, Bin Wu, Yuguo Zhen, Guixin Qin, Xuefeng Zhang, Natnael D. Aschalew, Zhe Sun, Tao Wang

**Affiliations:** ^1^ Key Laboratory of Animal Nutrition and Feed Science of Jilin Province, Key Laboratory of Animal Production Product Quality and Security Ministry of Education, JLAU‐Borui Dairy Science and Technology R&D Center, College of Animal Science and Technology Jilin Agricultural University Changchun China; ^2^ Institute of Animal and Veterinary Sciences Jilin Academy of Agricultural Sciences Changchun China; ^3^ Postdoctoral Scientific Research Workstation, Feed Engineering Technology Research Center of Jilin Province Changchun Borui Science & Technology Co., Ltd. Changchun China; ^4^ College of Agriculture and Environmental Science Dilla University Dilla Ethiopia; ^5^ College of Life Sciences, Engineering Research Center of Bioreactor and Pharmaceutical Development, Ministry of Education Jilin Agricultural University Changchun China

**Keywords:** gas chromatography–mass spectrometry, metabolites, milk, ultra‐high‐temperature sterilized

## Abstract

Processing can affect milk properties and alter the composition of milk metabolites, which has corresponding effects on milk flavor and quality. It is quite important to study the safe quality control of milk processing. Therefore, the purpose of this study was to identify metabolites at different steps of ultra‐high‐temperature‐sterilized (UHT) milk processing using gas chromatography–mass spectrometry (GC–MS). These steps included raw milk, pasteurized milk (80°C for 15 s), semi‐finished milk (after pasteurizing, it was homogenized at 75°C with pressure of 250 bar), UHT milk (at 140°C for 10 s), and finished milk (homogenized UHT milk). A total of 66 metabolites were identified across all samples, including 30 metabolites in the chloroform layers of the milk samples and 41 metabolites in the water layers; 5 metabolites were found in both layers. The metabolites were primarily fatty acids, amino acids, sugars, and organic acids. For example, pasteurized and ultra‐high‐temperature‐sterilized kinds of milk had lactose contents similar to those of raw milk, with increases in saturated fatty acids such as hexadecanoic acid and octadecanoic acid. Additionally, these findings indicated that these methods of processing can affect the contents of some components of milk. Therefore, from the perspective of milk's nutritional value and consumer health, the excessive heating of dairy products should be avoided and the milk heat treatment process should be standardized from the source.

## Graphical abstract

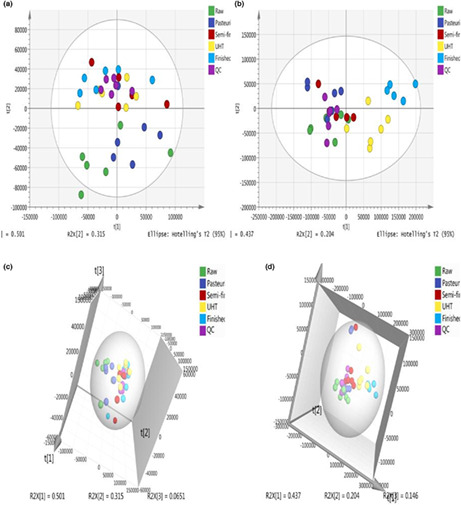

## 1 INTRODUCTION

Milk has high nutritional value and contains nutrients such as proteins, fats, lactose, essential fatty acids, vitamins, and enzymes that contribute to normal human growth, development, and body functions (Lordan et al., 2018). However, distinct conditions of processes will lead to different nutrient contents in milk or dairy products (Visioli & Strata, 2014). Correspondingly, different milk and dairy products have different effects on human health. Therefore, it is quite important to study the safe quality control of milk processing. Raw milk can easily be contaminated by spoilage microorganisms and food‐borne pathogens from various sources (soil, air, feed, water, equipment, or people). To ensure safety and increase shelf life, raw milk is routinely treated using processes such as pasteurization and ultra‐high‐temperature sterilization (UHT; Morales, 2003). Proper thermal processing can degrade non‐absorbable macromolecular proteins into digestible small‐molecular proteins and improve the nutritional value of milk. The effects of heating can affect milk properties and also alter the composition of milk metabolites, which has corresponding effects on milk flavor and quality (Caboni et al., 2019; Pereda et al., 2008; Yang et al., 2018). When the processing temperature exceeds 130°C, there will be biochemical changes in certain components of milk products (Sundekilde et al., 2013). Heating decomposes lactose into organic acids and encourages the formation of lactulose. Heating also causes the denaturation of whey proteins, the destruction of vitamins and enzymes, the hydrolysis of proteins and lipids, and the disturbance of calcium or phosphorus equilibrium (Dyer et al., 2016; Sakkas et al., 2014). During the storage of heated milk, the nutritional value loss is due to new substances formed by the Maillard reaction (Elliott et al., 2005). Unfortunately, it is very difficult to detect the biochemical processes of milk through common sensory and biochemical analyses in many practical applications.

Most of the studies focused on the effects of the different packaging and different storage conditions on the changes in milk composition, as well as the changes in the contents of special substances such as lactulose and furosine–lactulose in milk under different processing conditions (Bottiroli et al., 2021; Sukmaningrum et al., 2021; Tamime, 2009). However, less research has clarified the metabolites formed during the UHT treatment of milk. Metabolites directly reflect all the small‐molecular substances present in response to various factors or stimuli (Gowda & Djukovic, 2013; Sun et al., 2019), which can be used to dynamically analyze and monitor the changes in metabolites in the process (Tian et al., 2016). Gas chromatography–mass spectrometry (GC–MS) is the most commonly used analytical technique for the identification and quantitation of organic substances in complex matrices (He et al., 2008). Heat treatment is the most important way in dairy processing. From the perspective of the ability to kill microorganisms, the higher the heat‐treatment temperature of milk, the longer the time and the better the sterilization effect. However, from the perspective of milk quality, overheating may have a negative impact on milk, and even produce harmful substances. So, our hypothesis is overheating can affect the metabolites related to appearance, taste, and nutritional value of milk. Therefore, in this study, we used GC–MS to determine and compare metabolite differences during UHT milk processes. Thus, from the perspective of milk nutritional value and consumer health, it provides a basis for judging the potentially harmful substances in liquid milk and standardizes the milk thermal processing process from the source.

## 2 MATERIALS AND METHODS

### 2.1 Collection and preparation of milk samples

Six samples of bovine milk from each step of the processing of milk (raw, pasteurized, semi‐finished milk, sterilized, and finished milk) were collected at a dairy factory in Northeast China. The bovine milk was collected from healthy Holstein cows with similar body condition scores (3.25 ± 0.25), parity (2.5 ± 0.5 times), age (3.5 ± 0.5 years), and days of lactation (115 ± 5 days), as well as fed the same diet in October. The same samples of raw milk were submitted to pasteurization (80°C for 15 s), creating semi‐finished milk (after pasteurization, it was homogenized at 75°C with pressure of 250 bar), and then to ultra‐high‐temperature processing (at 140°C for 10 s), creating finished milk (homogenized UHT milk). All samples were transferred to sterilized tubes. The tubes were immediately cooled at 4°C after sampling and transported in thermal containers.

Each milk sample (15 mL) was transferred to a 50‐mL centrifuge tube and subjected to ultrasound for 15 min. After sonication, each sample was divided into six aliquots; each aliquot was 100 μL. To each aliquot, 250 μL of methanol and 125 μL of chloroform were added and the mixture was then vortexed for 1 min. Next, 380 μL of chloroform and 90 μL of aqueous potassium chloride (14.8 g/L) were added. After vortexing for another 1 min, samples were centrifuged at 16,000 *g* at 4°C for 10 min. From the supernatant of each sample, we collected 250 μL of the chloroform layer and 250 μL of the water‐phase layer and transferred these layers into separate Eppendorf tubes. After freeze drying for 24 h, 80 μL methoxypyridine solution (10 mg/ml) was added to each Eppendorf tube and we vortexed the tubes for 1 min. Each tube was then incubated at 37°C for 2 h to allow oximation. After incubation, 80 μL of N,O‐bis (trimethylsilane) trifluoroacetamide (BSTFA, containing 1% TMCS) was added to each tube and the tubes were vortexed for 1 min. Then, all tubes were incubated at 60°C for 1 h. After incubation, 100 μL samples were taken from all tubes for GC–MS analysis. In addition, 100 μL of each sample was mixed to prepare quality control (QC) samples. The QC sample processing was the same as that described above for the milk samples. The standard solution used was as follows: L‐asparagine (CCFD200711), lactose (CCFD200366), lactulose (CCFD200322), octadecanoic acid (CCHM700290), dodecanoic acid (CCFD200114), D(+)‐galactose (CCFD200078), hexadecanoic acid (CCFD200152), and tetradecanoic acid (CCHM510128); all the quality of samples was more than 99%, and the samples were purchased from National Standard Material Network. 1‐Octadecanol (CCEM500120) was used as the internal standard.

### 2.2 Linear relationship

The standard substance with a concentration of 1 g/mL was accurately absorbed and diluted into multiple working solutions with concentrations of 1, 2, 5, 10, 20, 50, and 100 μg/mL in turn for linear testing. A total of 1 mg samples in each treatment group were weighed and spiked with 0.1 mg/mL internal standard (1‐octadecanol). Each sample was added with 100 μL internal standard, and each sample was repeated three times. After adding internal standard, the samples were subjected to GC/MS analysis after derivatization.

### 2.3 GC conditions and MS methodology

The GC–MS analysis was performed using a TRACE 1310/TSQ 8000 Evo Gas Chromatograph Mass Spectrometer (Thermo Fisher Scientific, USA) equipped with a multipurpose sampler and an HP‐5MS (300 m × 0.25 mm × 0.25 μm) elastic quartz capillary column. The chromatographic conditions for the water layer and the chloroform layer were as follows: The GC temperature was initially 80°C for 1 min. Then, it was gradually increased in 6°C increments to 275°C, where it was held for 5 min. The injection volume of the sample was 1 μL, and the carrier gas used was helium at a flow rate of 1 mL/min; the split mode was 10:1. The MS transfer line temperature was 280°C. MS spectra were recorded in electron ionization mode (MS‐EI) with a 70 eV ionization energy, and the ion source temperature was set at 230°C. Full‐scan acquisition was used in the 50–550 amu range. Total ion currents were obtained after metabolomics analyses. The mass spectrograms were uniform at the chromatographic peaks, abundant, and with appropriate shapes; the spectra lacked undesirable phenomena such as oversaturation or tail dragging.

Compounds were identified based on their retention indices, mass spectral libraries, and authentic standard compound comparisons. Mass spectrometry was performed by comparing the mass spectra of unknown peaks with those stored in NIST2002 libraries. Kováts retention indices were determined by the injection of a solution containing the homologous series of normal alkanes (C10–C22) in a temperature‐programmed run (Van den Dool & Kratz, 1963), as described above. The missing value treatment was used in all samples and the comparison group samples. If the missing quantity of a substance was greater than 50% of the entire sample quantity, the substance was deleted and was not used in the data analysis. Otherwise, we filled in missing values with half the minimum value of the peak that was not missing for that compound.

### 2.4 Data analysis

The GC–MS data were processed via Pareto scaling, then imported into the SIMCA‐P + 14.0 software package (Umetrics) for statistical analysis. Principal component analysis (PCA) and orthogonal partial least‐squares discriminant analysis (OPLS‐DA) were used to visualize the metabolic alterations among samples after mean centering and unit variance scaling. Variable importance in the projection (VIP) ranks the overall contribution of each variable to the OPLS‐DA model and those metabolites with VIP >1.0. *p*‐Values < .05 were considered to represent significant differences among samples. Default cross‐validation was used to verify the results.

## 3 RESULTS

### 3.1 GC–MS analysis

In total, 66 metabolites across the categories of raw milk, pasteurized milk, semi‐finished milk, UHT milk, and finished milk samples were identified. Of these, 30 metabolites were identified in the chloroform layers of the milk samples (Table 1), while 41 were identified in the water layers (Table 2); 5 metabolites were found in both layers.

**TABLE 1:** Metabolites identified in the chloroform layers of the milk samples.

No.	Metabolite	RT/min^a^	RI^b^	Raw milk^c^	Pasteurized milk^c^	Semi‐finished milk^c^	UHT milk^c^	Finished milk^c^
1	Benzene	6.21	1074	9.11 × 10^6^	9.58 × 10^6^	1.03 × 10^7^	9.09 × 10^6^	9.40 × 10^6^
2	Ethanol	6.57	1086	3.70 × 10^7^	3.92 × 10^7^	4.15 × 10^7^	4.25 × 10^7^	4.41 × 10^7^
3	Nonane	7.79	1126	8.78 × 10^6^	9.88 × 10^6^	1.25 × 10^7^	1.23 × 10^7^	1.35 × 10^7^
4	Pyridine	8.42	1147	2.14 × 10^7^	2.16 × 10^7^	2.44 × 10^7^	2.25 × 10^7^	2.35 × 10^7^
5	Undecane	9.03	1168	7.37 × 10^7^	8.92 × 10^7^	9.70 × 10^7^	1.03 × 10^8^	1.08 × 10^8^
6	Decane	10.42	1214	7.26 × 10^6^	8.58 × 10^6^	1.15 × 10^7^	9.80 × 10^6^	1.24 × 10^7^
7	Pentasiloxane	11.75	1258	9.85 × 10^6^	9.70 × 10^6^	9.97 × 10^6^	8.42 × 10^6^	7.71 × 10^6^
8	Dodecane	12.84	1295	9.31 × 10^6^	8.72 × 10^6^	1.09 × 10^7^	1.04 × 10^7^	1.24 × 10^7^
9	2,6‐Dimethyl‐undecane	12.91	1297	1.57 × 10^7^	1.66 × 10^7^	2.11 × 10^7^	2.05 × 10^7^	2.38 × 10^7^
10	Tetradecane	13.48	1319	0	7.03 × 10^6^	1.38 × 10^7^	1.34 × 10^7^	1.57 × 10^7^
11	Urea	13.52	1321	8.27 × 10^7^	8.48 × 10^7^	1.12 × 10^8^	1.02 × 10^8^	1.12 × 10^8^
12	Pentadecane	13.6	1324	1.07 × 10^7^	1.14 × 10^7^	1.47 × 10^7^	1.39 × 10^7^	1.62 × 10^7^
13	2,4‐Dimethyldodecane	13.92	1337	3.79 × 10^7^	4.03 × 10^7^	5.17 × 10^7^	5.09 × 10^7^	5.58 × 10^7^
14	Glycerol	14.05	1342	1.71 × 10^8^	1.91 × 10^8^	2.32 × 10^8^	2.34 × 10^8^	2.62 × 10^8^
15	Hexadecane	16.72	1461	1.42 × 10^7^	1.57 × 10^7^	2.04 × 10^7^	2.27 × 10^7^	1.26 × 10^7^
16	Heptadecane	17.85	1518	3.07 × 10^7^	3.30 × 10^7^	4.32 × 10^7^	4.19 × 10^7^	5.23 × 10^7^
17	Propane	18	1525	9.11 × 10^7^	9.79 × 10^7^	1.22 × 10^8^	1.19 × 10^8^	1.31 × 10^8^
18	Butylated hydroxytoluene	18.26	1538	1.84 × 10^7^	1.87 × 10^7^	3.13 × 10^7^	2.89 × 10^7^	3.27 × 10^7^
19	Dodecanoic acid	20.4	1645	5.23 × 10^7^	4.60 × 10^7^	3.84 × 10^7^	4.45 × 10^7^	0
20	1,2,3‐Propanetricarboxylic acid	22.57	1771	4.48 × 10^7^	4.42 × 10^7^	8.55 × 10^7^	2.55 × 10^7^	3.57 × 10^7^
21	Tetradecanoic acid	23.08	1804	2.55 × 10^8^	2.31 × 10^8^	2.37 × 10^8^	2.35 × 10^8^	2.22 × 10^8^
22	2‐Methyl‐eicosane	23.87	1844	8.04 × 10^7^	8.64 × 10^7^	9.86 × 10^7^	9.83 × 10^7^	1.07 × 10^8^
23	Hexadecanoic acid	25.52	1935	4.31 × 10^9^	4.62 × 10^9^	5.13 × 10^9^	5.12 × 10^9^	5.26 × 10^9^
24	Trans‐9‐octadecenoic acid	27.47	2065	9.39 × 10^8^	1.00 × 10^9^	9.65 × 10^8^	9.34 × 10^8^	8.96 × 10^8^
25	Trans‐13‐octadecenoic acid	27.55	2070	1.25 × 10^8^	1.19 × 10^8^	8.49 × 10^7^	9.61 × 10^7^	1.25 × 10^8^
26	Octadecanoic acid	27.75	2083	2.16 × 10^9^	2.43 × 10^9^	2.78 × 10^9^	2.79 × 10^9^	2.98 × 10^9^
27	Monopalmitoylglycerol	30.85	2290	1.14 × 10^8^	7.50 × 10^7^	0	0	0
28	D‐(+)‐cellobiose	31.97	2365	2.16 × 10^9^	3.63 × 10^9^	3.52 × 10^9^	2.41 × 10^9^	2.11 × 10^9^
29	D‐lactose	32.16	2377	4.74 × 10^8^	7.78 × 10^8^	1.06 × 10^9^	5.12 × 10^8^	5.25 × 10^8^
30	Cholesterol	39.68	2847	6.31 × 10^8^	8.24 × 10^8^	1.03 × 10^9^	1.01 × 10^9^	6.81 × 10^8^

^a^ Retention time on HP‐5MS column.

^b^ Retention indices calculated on HP‐5MS column against n‐alkanes.

^c^ Data indicate the abundance of peak areas per sample; each value is reported as the mean of six replicates from each treatment (*n* = 6).

**TABLE 2:** Metabolites identified in the water layers of the milk samples.

No.	Metabolite	RT/min^a^	RI^b^	Raw milk^c^	Pasteurized milk^c^	Semi‐finished milk^c^	UHT milk^c^	Finished milk^c^
1	1,2‐Butanediol	6.03	1068	1.47 × 10^7^	0	1.26 × 10^7^	1.67 × 10^7^	1.63 × 10^7^
2	Urea	6.34	1078	5.48 × 10^9^	4.58 × 10^9^	5.35 × 10^9^	7.24 × 10^9^	5.98 × 10^9^
3	4,6‐Dimethyl‐dodecane	6.42	1081	3.04 × 10^7^	2.39 × 10^7^	2.58 × 10^7^	1.90 × 10^7^	2.33 × 10^7^
4	L‐proline	6.92	1097	4.19 × 10^7^	3.05 × 10^7^	3.71 × 10^7^	4.68 × 10^7^	3.96 × 10^7^
5	Glycine	6.99	1100	6.44 × 10^7^	6.68 × 10^7^	9.05 × 10^7^	1.40 × 10^8^	1.24 × 10^8^
6	Butanedioic acid	7.09	1103	4.09 × 10^7^	3.14 × 10^7^	5.04 × 10^7^	6.78 × 10^6^	6.13 × 10^7^
7	Propanoic acid	7.22	1107	5.82 × 10^7^	4.49 × 10^7^	3.97 × 10^7^	5.02 × 10^7^	4.37 × 10^7^
8	Aminomalonic acid	8.9	1163	0	0	4.29 × 10^7^	1.05 × 10^8^	9.11 × 10^7^
9	L‐aspartic acid	9.46	1182	3.26 × 10^7^	3.45 × 10^7^	4.14 × 10^7^	5.76 × 10^7^	5.35 × 10^7^
10	Pentanedioic acid	10.08	1203	1.01 × 10^8^	8.28 × 10^7^	1.01 × 10^8^	1.27 × 10^8^	1.16 × 10^8^
11	Glutamic acid	10.67	1222	4.22 × 10^8^	3.27 × 10^8^	3.77 × 10^8^	4.87 × 10^8^	4.28 × 10^8^
12	D‐(+)‐xylose	11.07	1236	0	0	0	2.36 × 10^7^	2.87 × 10^7^
13	D‐arabinose	11.08	1236	5.47 × 10^7^	4.34 × 10^7^	3.96 × 10^7^	4.46 × 10^7^	4.65 × 10^7^
14	D‐ribose	11.41	1247	1.16 × 10^8^	9.25 × 10^7^	8.41 × 10^7^	1.06 × 10^8^	1.13 × 10^8^
15	2‐Methyl‐nonadecane	12.02	1267	6.27 × 10^7^	5.60 × 10^7^	5.97 × 10^7^	7.72 × 10^7^	7.93 × 10^7^
16	D‐(−)‐rhamnose	12.21	1274	8.96 × 10^7^	7.17 × 10^7^	7.01 × 10^7^	9.18 × 10^7^	9.45 × 10^7^
17	4‐Pyrimidinecarboxylic acid	12.65	1288	2.81 × 10^8^	2.47 × 10^8^	3.25 × 10^8^	5.03 × 10^8^	4.44 × 10^8^
18	1‐Propene‐1,2,3‐tricarboxylic acid	12.72	1291	4.05 × 10^7^	2.80 × 10^7^	4.22 × 10^7^	6.83 × 10^7^	7.01 × 10^7^
19	Phosphoric acid	12.86	1295	3.11 × 10^8^	2.64 × 10^8^	3.71 × 10^8^	5.6 × 10^8^	6.44 × 10^8^
20	D‐(−)‐lyxofuranose	12.97	1299	0	0	3.54 × 10^7^	3.53 × 10^7^	7.31 × 10^7^
21	1,2,3‐Propanetricarboxylic acid	13.87	1335	7.18 × 10^9^	6.43 × 10^9^	7.12 × 10^9^	9.13 × 10^9^	9.12 × 10^9^
22	D‐galactopyranose	14.09	1344	1.73 × 10^8^	1.34 × 10^9^	1.75 × 10^9^	2.04 × 10^9^	9.12 × 10^9^
23	L‐asparagine	14.25	1350	4.74 × 10^8^	3.39 × 10^8^	4.53 × 10^8^	6.16 × 10^8^	1.90 × 10^9^
24	D‐mannose	14.88	1375	2.60 × 10^8^	2.30 × 10^8^	1.79 × 10^8^	2.12 × 10^8^	5.44 × 10^8^
25	D‐galactose	14.96	1378	1.35 × 10^9^	1.22 × 10^9^	1.09 × 10^9^	1.36 × 10^9^	2.16 × 10^8^
26	D‐glucose	15.06	1382	1.66 × 10^9^	1.35 × 10^9^	1.40 × 10^9^	1.86 × 10^9^	1.63 × 10^9^
27	DL‐lyxopyranose	15.22	1389	4.87 × 10^7^	3.81 × 10^7^	4.75 × 10^7^	5.67 × 10^7^	1.94 × 10^9^
28	Lactulose	16.14	1432	3.05 × 10^7^	1.75 × 10^7^	1.92 × 10^7^	1.93 × 10^7^	9.05 × 10^7^
29	D‐(+)‐talopyranose	16.5	1450	1.70 × 10^8^	1.91 × 10^7^	1.26 × 10^8^	1.27 × 10^8^	1.67 × 10^7^
30	Ribonic acid	16.74	1462	5.18 × 10^7^	4.59 × 10^7^	3.55 × 10^7^	2.84 × 10^7^	2.01 × 10^8^
31	N‐acetyl‐D‐glucosamine	18.08	1529	4.86 × 10^8^	4.69 × 10^8^	4.18 × 10^8^	5.66 × 10^8^	2.75 × 10^9^
32	Myo‐inositol	18.21	1536	5.06 × 10^8^	4.16 × 10^8^	4.60 × 10^8^	6.01 × 10^8^	5.98 × 10^8^
33	Uric acid	18.5	1550	1.80 × 10^8^	1.47 × 10^8^	1.65 × 10^8^	2.15 × 10^8^	6.41 × 10^8^
34	Octadecanoic acid	20.74	1662	1.00 × 10^9^	1.15 × 10^9^	1.06 × 10^9^	1.42 × 10^9^	1.95 × 10^8^
35	Eicosane	25.81	1954	1.96 × 10^7^	2.29 × 10^7^	1.24 × 10^7^	1.59 × 10^7^	1.69 × 10^7^
36	Monopalmitoylglycerol	26.19	1979	1.65 × 10^7^	2.27 × 10^7^	1.83 × 10^7^	2.46 × 10^7^	1.16 × 10^7^
37	1,5‐Anhydro‐D‐sorbitol	26.79	2010	3.72 × 10^7^	2.38 × 10^7^	2.16 × 10^7^	3.15 × 10^7^	2.54 × 10^7^
38	D‐(+)‐cellobiose	28.97	2082	5.89 × 10^9^	8.60 × 10^9^	8.18 × 10^9^	8.01 × 10^9^	1.32 × 10^7^
39	D‐mannopyranose	30.17	2127	16.2 × 10^9^	16.9 × 10^9^	17.1 × 10^9^	18.5 × 10^9^	8.00 × 10^9^
40	D‐lactose	30.79	2152	5.3 × 10^9^	6.78 × 10^9^	7.65 × 10^9^	8.22 × 10^9^	21.2 × 10^9^
41	Galactinol	33.66	2242	8.33 × 10^7^	9.36 × 10^7^	1.09 × 10^8^	1.10 × 10^8^	7.58 × 10^9^

^a^ Retention indices calculated on HP‐5MS column against n‐alkanes.

^b^ Data indicate the abundance of peak areas per sample; each value is reported as the mean of six replicates from each treatment (*n* = 6).

^c^ Retention time on HP‐5MS column.

### 3.2 Analysis of the different metabolites

**FIGURE 1:**
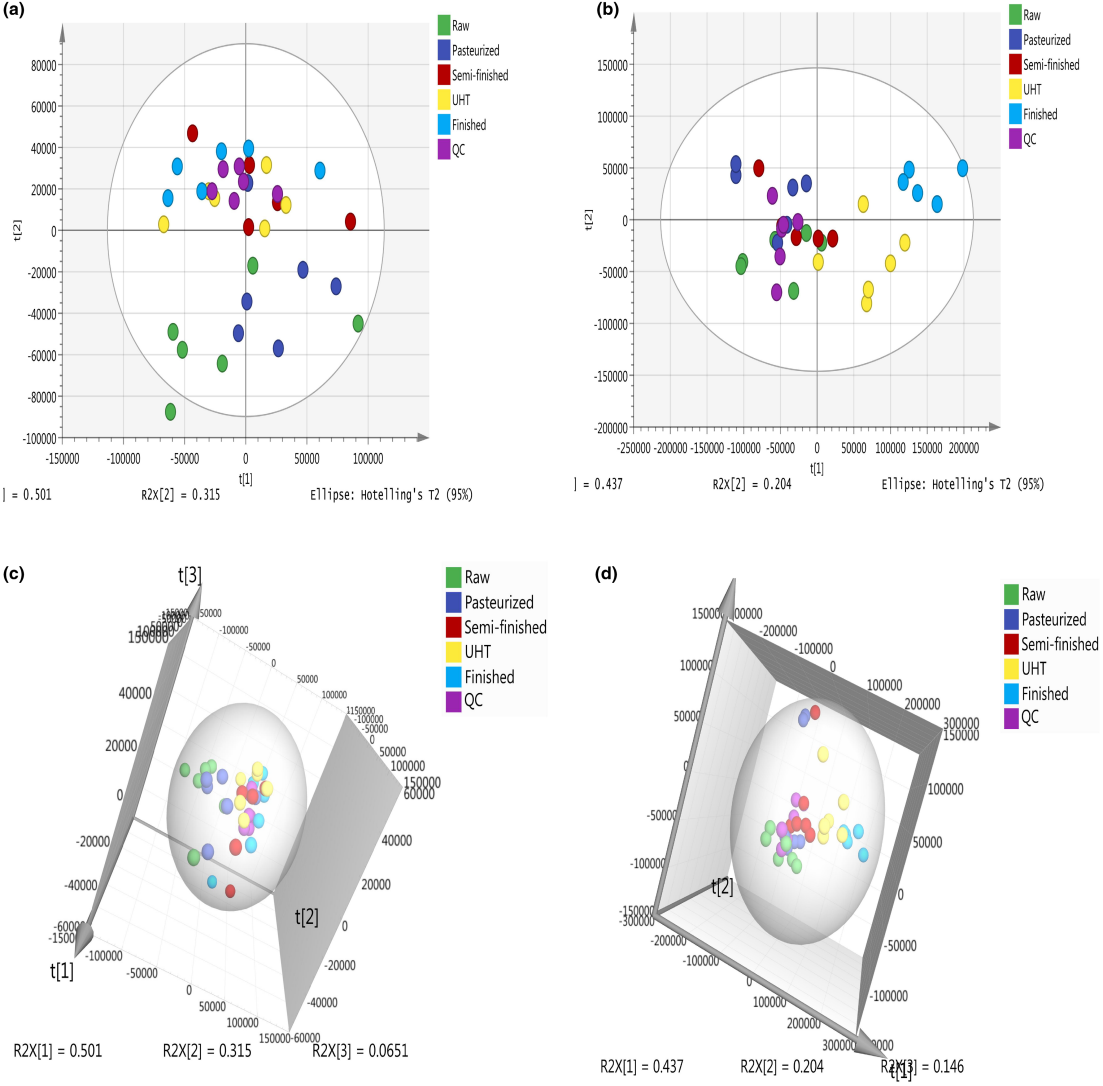
Principal component analysis (PCA) of the milk metabolites. (a) Two‐dimensional PCA of milk chloroform layer; (b) two‐dimensional PCA of milk water layer; (c) three‐dimensional PCA of milk chloroform layer; and (d) three‐dimensional PCA of milk water layer.

The PCA score plot of the metabolic profiles showed significantly separated clusters among the different steps of UHT milk processing in the chloroform (Figure 1a,c) and water layers (Figure 1b,d). All score plots for the samples were in the Hotelling T2 ellipse with 95% confidence. The PCA showed high repeatability across biological replicates (Nousiainen et al., 2004). Generally, the QC samples in the chloroform layer (Figure 1a,c) of each treatment of milk were closely clustered together. While the QC samples in the water layer (Figure 1b,d) were relatively clustered together.

**FIGURE 2:**
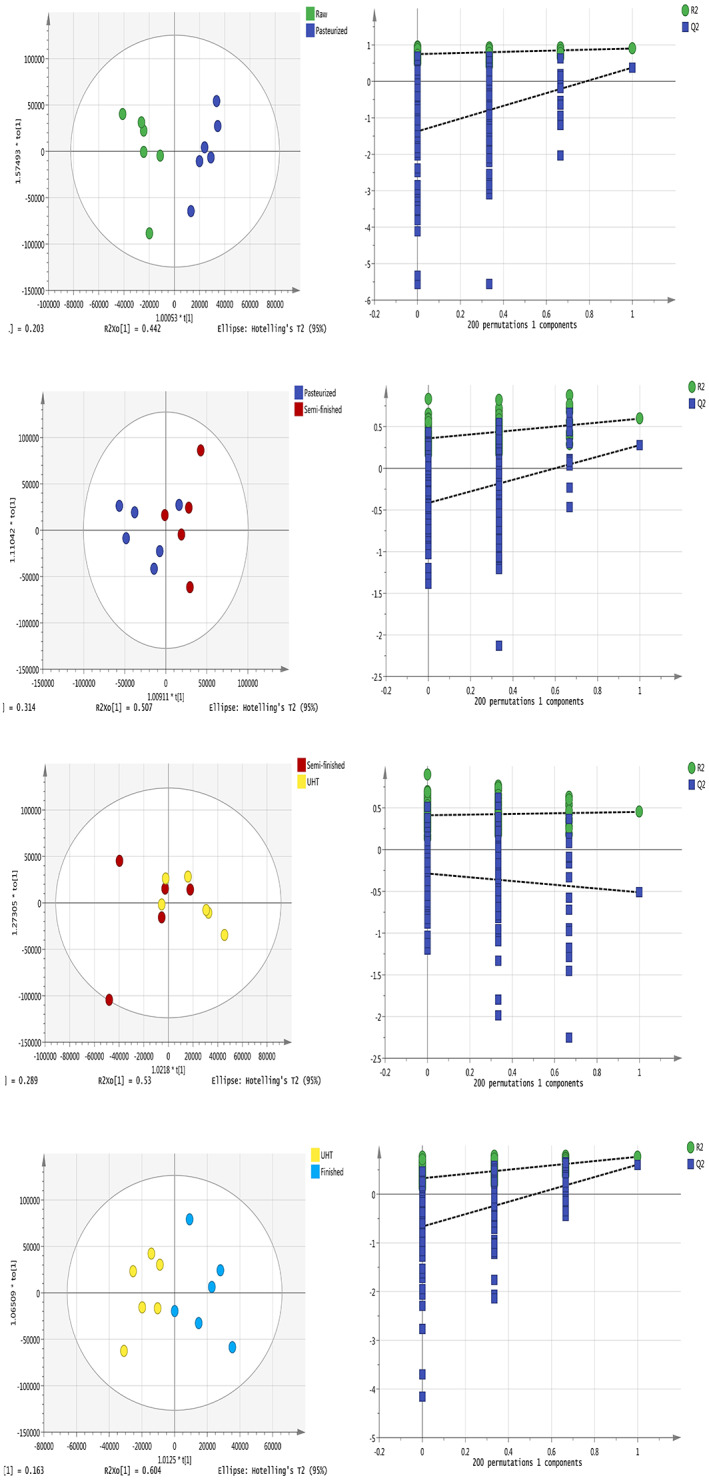
Orthogonal partial least squares discriminant analyses (OPLS‐DAs) of metabolites in the chloroform layers of milk samples (left) and cross‐validation conducted using permutation tests (*n* = 200) for the milk chloroform layer (right).

**FIGURE 3:**
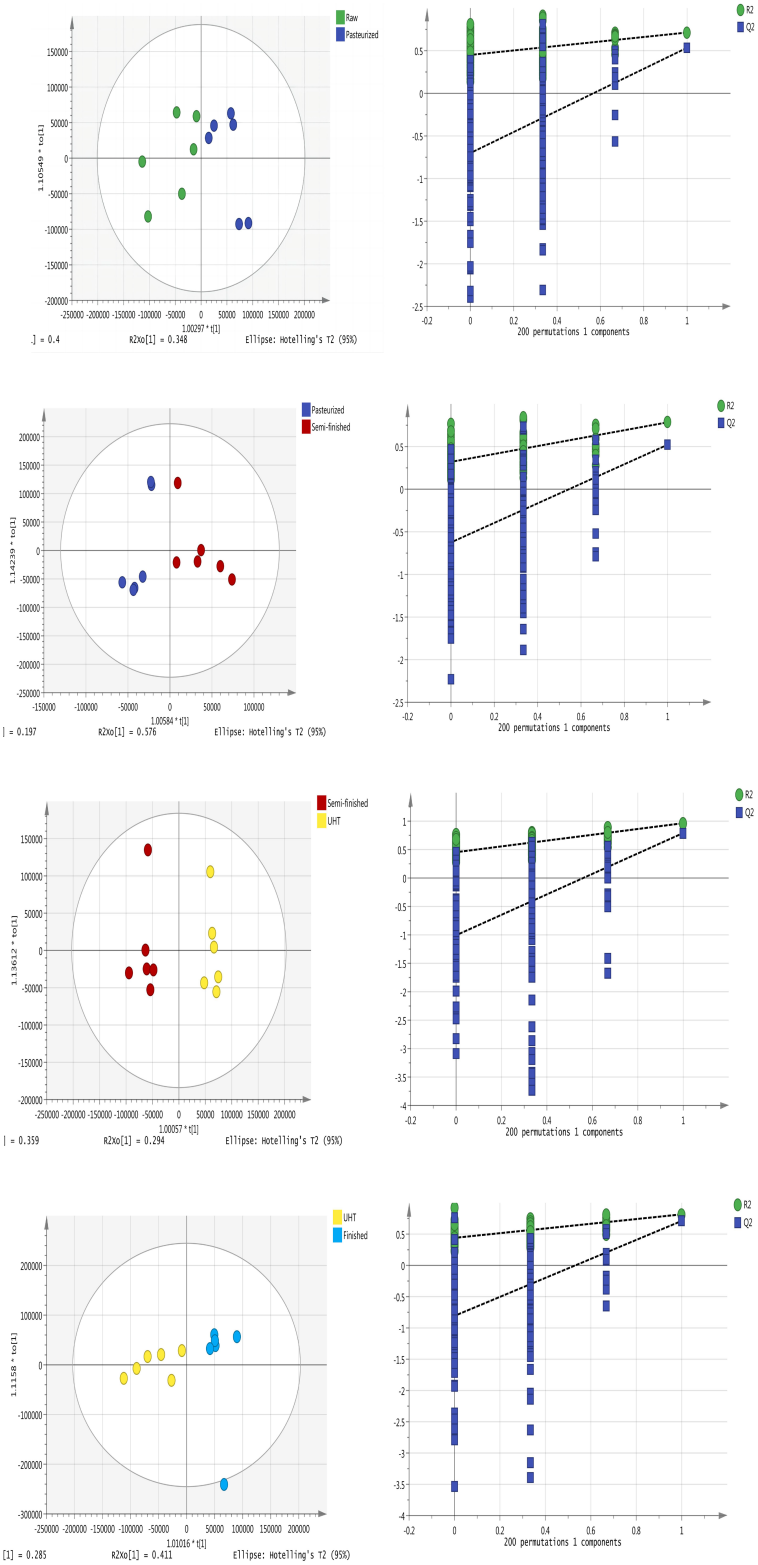
Orthogonal partial least squares discriminant analyses (OPLS‐DAs) of metabolites in the water layers of milk samples (left) and cross‐validation conducted using permutation tests (*n* = 200) for the milk water layer (right).

To further investigate the separation between adjacent processing durations, orthogonal partial least squares discriminant analyses (OPLS‐DAs) showed the separation of metabolites in the chloroform layers of milk samples collected at different processing steps. In the OPLS‐DA model, the chloroform‐layer samples from each milk type were well separated, indicating that there were differences in the milk components between adjacent types (Figure 2). Similarly, the OPLS‐DA analysis indicated that the water‐layer milk metabolites differed significantly between adjacent milk types and that replacement verification met the conditions (Figure 3). The *R* ^2^ and *Q* ^2^ were more than 0.5, meaning that the OPLS‐DA model was sTABLE and reliable. The evaluation parameters of the OPLS‐DA models are given in the Supplementary Material (Tables S1 and S2). The OPLS‐DA model in both layers was explored using a permutation test (*n* = 200). The results of the permutation test show that the slope of the regression line in the FIGURE is larger, indicating that the models of the two groups were successfully established.

### 3.3 Identification of differing metabolites

**FIGURE 4:**
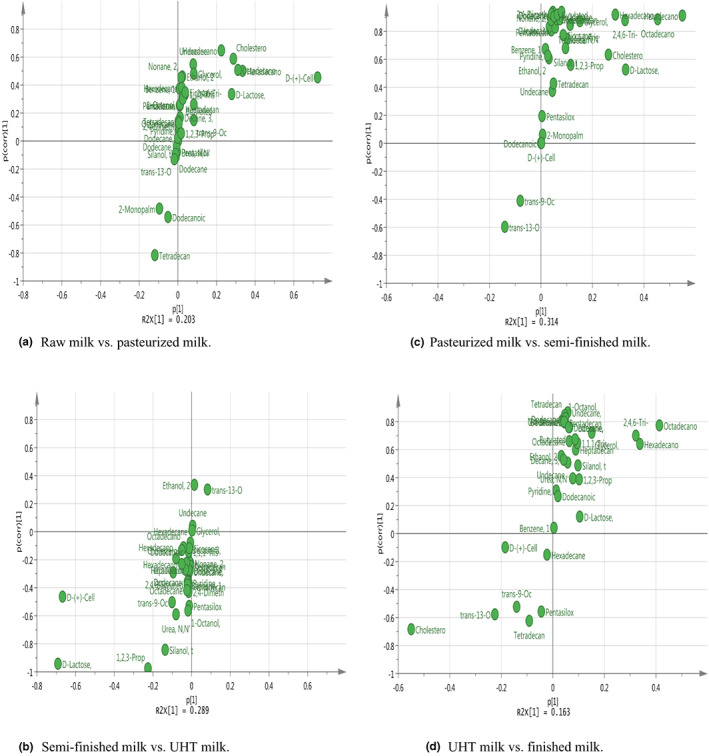
S‐plot in the chloroform layer differing among samples. (a) Raw milk versus pasteurized milk; (b) pasteurized milk versus semi‐finished milk; (c) semi‐finished milk versus UHT milk; and (d) UHT milk versus finished milk.

**FIGURE 5:**
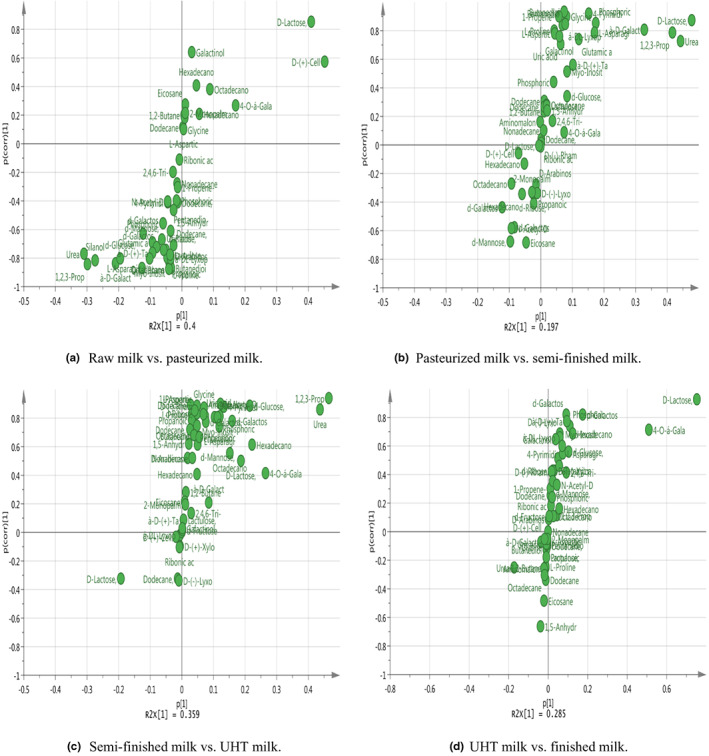
S‐plot in the water layer differing among samples. (a) Raw milk versus pasteurized milk; (b) pasteurized milk versus semi‐finished milk; (c) semi‐finished milk versus UHT milk; and (d) UHT milk versus finished milk.

A clear separation in the metabolomic profiles was observed between different steps of UHT milk processing. S‐plots showing the key metabolites are given in Figures 4 and 5. On the left and right side of the S‐plot, the variables with strong model contribution and high statistical reliability were screened in order to uncover potential biomarkers to characterize the metabolic discriminations between raw milk and pasteurized milk, pasteurized milk and semi‐finished milk, semi‐finished milk and ultra‐high‐temperature‐sterilized (UHT) milk, and UHT milk and finished milk. In total, 10 differentially expressed metabolites were identified among the different steps of UHT milk processing (VIP >1, *p* < .05), as indicated in Table 3.

**TABLE 3:** Potential biomarkers of milk in processing.

Treatment	No.	Compounds	RT/min^a^	VIP	*p* value
Raw milk vs. pasteurized milk	1	Hexadecanoic acid	31.17	1.17	.032
	2	Tetradecanoic acid	20.74	1.89	.041
	3	D‐lactose	32.36	2.67	.005
Pasteurized milk vs. semi‐finished milk	1	Octadecanoic acid	27.75	2.48	.029
	2	Hexadecanoic acid	31.17	1.53	.002
	3	D‐lactose	32.36	2.78	.001
	4	D‐galactose	14.96	2.01	.008
	5	L‐asparagine	14.25	1.06	.011
Semi‐finished milk vs. UHT milk	1	Hexadecanoic acid	31.17	1.66	.028
	2	Octadecanoic acid	27.75	1.19	.049
	3	D‐lactose	32.36	3.63	.038
UHT milk vs. finished milk	1	Hexadecanoic acid	31.17	1.30	.003
	2	Octadecanoic acid	27.75	1.74	.032
	3	D‐lactose	32.36	4.62	.001
	4	Lactulose	16.14	1.94	.000
	5	D‐galactose	14.96	1.21	.000
	6	Dodecanoic acid	20.41	1.06	.002

^a^ Retention time on HP‐5MS column. In the chloroform and water layers of the milk: D‐lactose. In the chloroform layers of the milk: hexadecanoic acid, tetradecanoic acid, L‐asparagine, and dodecanoic acid. In the water layers of the milk: octadecanoic acid, D‐galactose, and lactulose.

**FIGURE 6:**
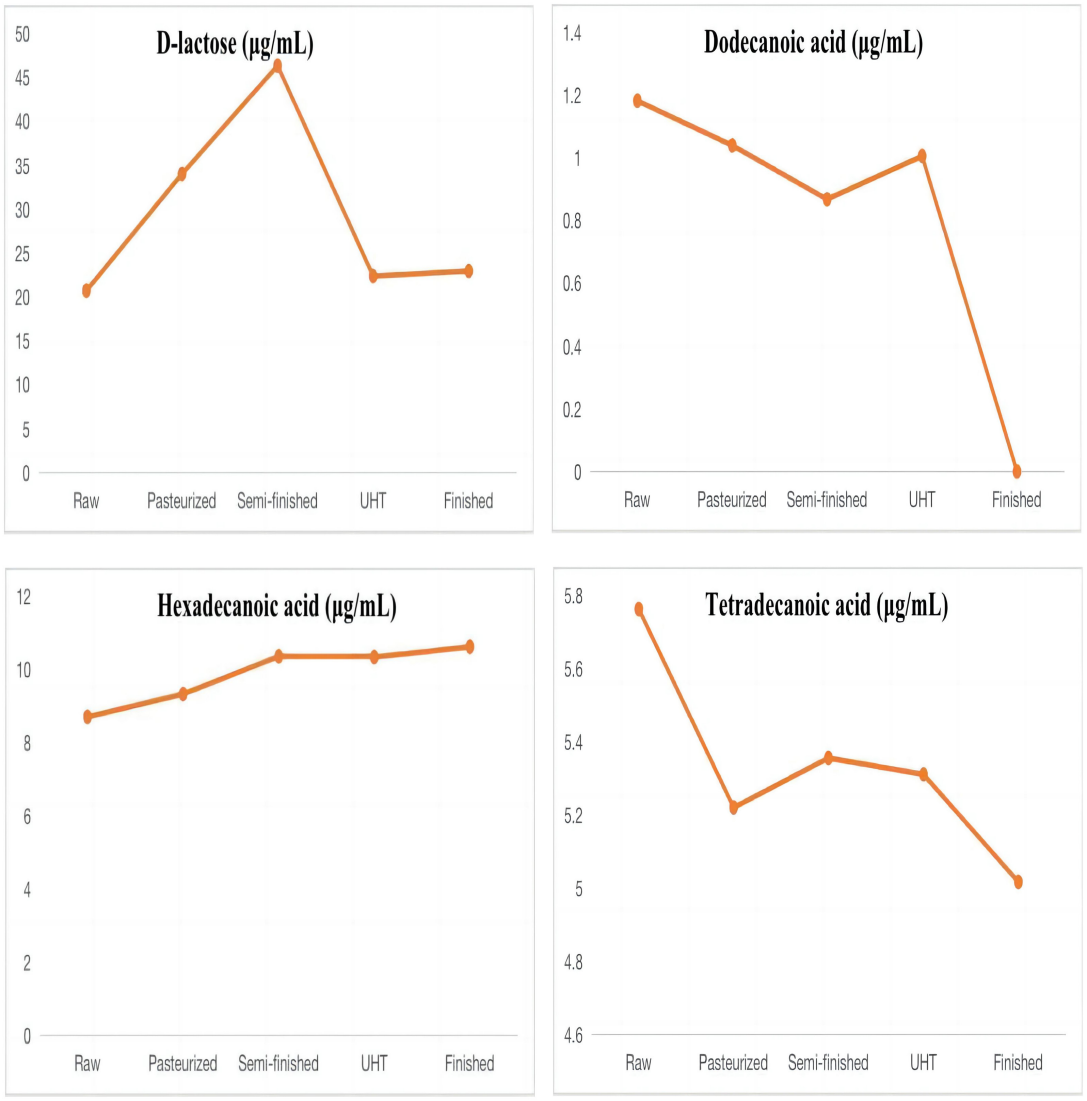
Main metabolites differing among milk samples in the chloroform layer.

**FIGURE 7:**
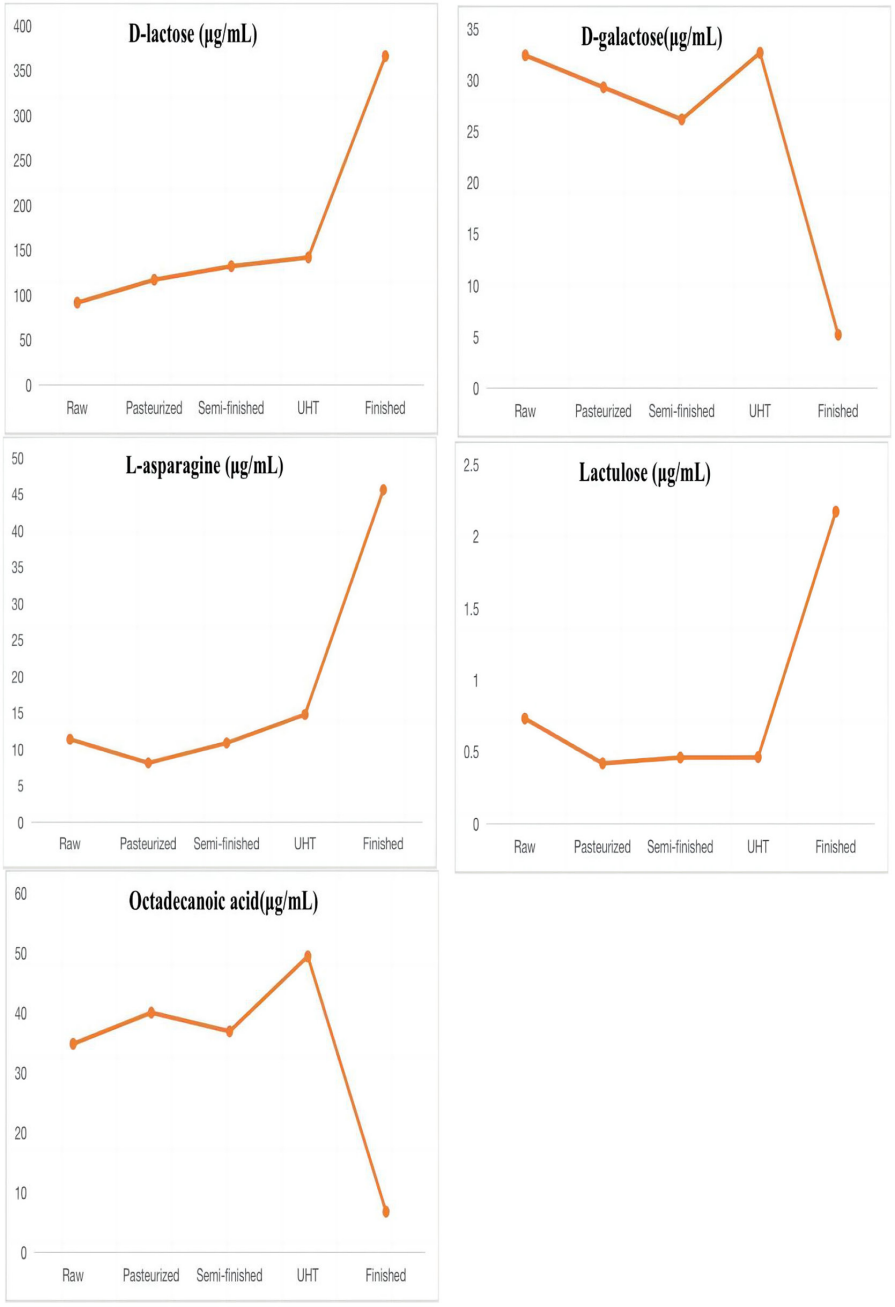
Main metabolites differing among milk samples in the water layer.

The result of linearity is shown in Table 4. The method gave a good range of linearity. In addition, in the chloroform layers of the milk, standard recovery rate of hexadecanoic acid, tetradecanoic acid, dodecanoic acid, and D‐lactose were 101.3%, 99.8%, 102.4%, and 100.3%, respectively. In the water layers of the milk, standard recovery rate of L‐asparagine, octadecanoic acid, D‐galactose, lactulose, and D‐lactose were 100.5%, 101.3%, 100.1%, 97.3%, and 98.6%, respectively. The metabolites that differed between raw milk and pasteurized milk were hexadecanoic acid, tetradecanoic acid, and D‐lactose. The metabolites that differed between pasteurized milk and semi‐finished milk were octadecanoic acid, hexadecanoic acid, D‐lactose, D‐galactose, and L‐asparagine. The metabolites that differed between semi‐finished milk and UHT milk were hexadecanoic acid, octadecanoic acid, and D‐lactose. The metabolites that differed between UHT milk and the finished milk were hexadecanoic acid, octadecanoic acid, D‐lactose, lactulose, D‐galactose, and dodecanoic acid. The content of organic acids such as hexadecanoic acid, tetradecanoic acid, and octadecanoic acid decreased with the increase in heating temperature. Lactulose content showed a significant increasing trend, while the D‐galactose content showed a decreasing trend in the water layer. Lactose first increased and then decreased in the chloroform layer. The main variations in metabolites are shown in Figures 6 and 7.

**TABLE 4:** Linearity and minimum method detection limits.

Compounds	Linear equations	*R* ^2^
L‐asparagine	*y* = 2.788026E−005 *x* + 7.926463E‐005	.998
D‐galactose	*y* = 2.81702 *x* + 2.729763	.999
D‐lactose	*y* = 1.734829 *x* − 0.459496	.997
Lactulose	*y* = 2.331610 *x* + 3.533199	.998
Octadecanoic acid	*y* = 0.138605 *x* − 0.099478	.997
Hexadecanoic acid	*y* = 0.294355 *x* − 0.266608	.997
Tetradecanoic acid	*y* = 0.475958 *x* − 0.405386	.997
Dodecanoic acid	*y* = 0.559644 *x* − 0.303804	.997

## 4 DISCUSSION

Metabolomic analysis is a direct, objective, and accurate method that describes biological processes (Gowda & Djukovic, 2013). We used a metabolomics approach (GC–MS) to assess changes in metabolites among samples of milk subjected to different degrees of processing. In total, 66 metabolites were identified in our study; these were mainly classified as fatty acids, sugars, and organic acids. Raw, pasteurized, and UHT kinds of milk had very similar fatty acid profiles. Pestana et al. (2015) also suggested that the treatments conducted during the pasteurization and commercial sterilization processes in milk had little effect on the fatty acid profile.

Raw milk, similar to pasteurization and UHT milk, reserved lactose. The composition of the milk was slightly altered with sterilization and ultra‐high‐temperature processes, increasing the contents of fatty acids and urea. Studies have shown that the organic acids produced by the degradation of lactose in milk thermal processing are mainly formic acid, and also produce lactic acid, propionic acid, butyric acid, acetic acid, etc. In addition, lactose did not change when heated to 100°C, and only these volatile organic acids were produced when heated above 100°C (Cho et al., 2012; Montilla et al., 1996). Our study found that the D‐lactose content increased significantly may be due to the evaporation of water during the raw milk to pasteurization (80℃). But, until the temperature is higher than 100 °C, the content of lactose begins to decrease because of the decomposition of itself.

When semi‐finished milk is processed into UHT milk, lactose undergoes three reactions: isomerization, hydrolysis, and the Maillard reaction. These reactions take place in a dynamic equilibrium (Choi et al., 1994). Lactose is formed when glucose and galactose are connected by a 1,4‐glycosidic bond. The increase in glucose and galactose contents is due to the hydrolysis of lactose, which may also be caused by the decrease in lactose content as the temperature rises (Elisabeth et al., 2020; Li et al., 2019).

Milk fat is mainly composed of fatty acids. Our results indicated that the concentrations of octadecanoic acid and hexadecanoic acid were increased by processing (Dave et al., 2002). Similar results were obtained in Brazilian dairy products, including raw, pasteurized, and UHT bovine milk, as described by Pestana et al. (2015) and Nunes and Torres (2010). This might be because lipase hydrolysis in the milk fat is involved in lipolysis in UHT milk, and lipase hydrolysis releases short‐ and long‐chain fatty acids (Loften et al., 2014). Therefore, during heat treatment, fat is likely hydrolyzed, increasing the contents of palmitate and stearic acid. As fatty acids are susceptible to oxidative rancidity, tetradecanoic acid is unstable, and the levels of this acid decrease during pasteurization (80°C for 15 s). Similarly, previous studies have demonstrated that heating milk to 120°C reduces the content of cholesterol fatty acids, such as dodecanoic acid and myristic acid, but that heat treatment at 75°C has no effect on these acids (Speziali et al., 2018). In addition, dodecanoic acid may inhibit cholesterol deposition and improve gastrointestinal immune function (Sakkas et al., 2014). However, dodecanoic acid disappears after UHT processing, decreasing the available nutrients in this type of milk. This is also consistent with the reports of Pestana et al. (2015). In one study, dodecanoic acid did not show differences after pasteurization and UHT processing, but it may have antiviral and antibacterial functions and might act as an anti‐caries and anti‐plaque agent (Claeys et al., 2014). In summary, our results showed that the abundances of various milk metabolites differed among the UHT milk steps, suggesting that the increase in milk temperature during processing might change the structure and abundance of milk components.

Lactulose is a disaccharide composed of galactose and fructose, which is a synthetic form of the base isomerization of lactose under the catalysis of free amino groups of casein during milk heat treatment (Claeys et al., 2002). It is used as an analytical indicator to distinguish pasteurized milk from UHT milk (Feinberg et al., 2006). The content of lactulose in pasteurized processing milk increased as the sterilization temperature and sterilization time rose. Researchers found that when pasteurized, the content of lactulose in milk linearly increased with the increase in sterilization temperature (85, 90, 95, and 100°C) and time (15, 30, 45, 75, and 90 s). Additionally, the impact of sterilization temperature is more crucial than that of sterilization time. While processed at ultra‐high heat, the content of lactulose in milk was found to increase along with the sterilization temperature (137–142°C) and sterilization time (2, 5, and 15 s) (Huang, 2007). While the results of our study were consistent with these findings, the specific change rule of lactulose in pasteurized milk with temperature and time is still unclear (De Block et al., 1996). In addition, in our study, as lactulose synthesis progressed, the content of galactose, which is synthetic lactulose, decreased. In addition, as lactulose synthesis progressed, the content of galactose, which is synthetic lactulose, decreased.

Elliott and Deeth (2002) studied the thermal damage of raw milk, UHT milk, and Pasteur milk and found that lactose is the most reliable indicator of the heat treatment process of milk because it is hardly affected by cold storage after UHT treatment. The glucose unit of lactose is partially isomerized to lactose when heated, and it is not an inherent component of milk. The amount of lactose can reflect the intensity or duration of heating. Furosine and furfural products of the Maillard reaction are used as specific indicators of the effect of heating treatments on milk quality (Ferrer et al., 2000; Pellegrino et al., 1995). Furosine is an analytical artifact formed by acid hydrolysis of the Amadori compound and can be used as an indicator of the Maillard reaction. Determination of furosine in heated milk as a measure of heat intensity during processing. Furosine is not present in heat‐treated milk but it is an artifact of the method in order to detect lactulose‐lysine. Due to the acid hydrolysis of ε‐deoxyketosyl compounds, furosine is formed (Pizzoferrato et al., 1998; Roland & Jan, 1996).

Furthermore, under the catalysis of acid, alkali, or alkaline earth metal ions, the glucose on the lactose unit is directly isomerized to the product sugar, thereby producing lactulose, and in the presence of oxygen, the olefinic diol double bond is cleaved to form the corresponding carboxylic acids, such as formic acid and gum acid. Additionally, dicarbonyl products formed from sugar degradation are rearranged by diphenylglycolic acid to form various organic acids; these may lead to changes in the experimental results obtained for organic acids (Fox et al., 2015). Lactose or lactulose can also be further degraded by the Maillard reaction with casein to form fructosyl lysine. However, these reaction products are very small in milk, and their production is mainly determined by the degree of thermal processing (O'Brien, 2009).

In addition, the contents of milk fat tend to increase when milk is stored, meaning that homogenization is required during milk processing. Homogenization primarily reduces the diameter of milk fat globules, decreasing the fat volume and suppressing the rise in the number of fat globules. This process also allows fat to be more easily absorbed in the process of human digestion (Ren et al., 2019). Homogenization and heat treatment also increase the contents of glycosylated molecules and denatured milk proteins to stabilize these fat globules (Aktağ et al., 2019). However, in our study, no substances related to homogenization were identified across any of the samples. Thus, it is unclear whether the observed changes in nutritional components among milk types were related to homogenization.

## 5 CONCLUSIONS

This study demonstrated that the metabolites of milk were slightly altered by ultra‐high‐temperature processes. Pasteurized and ultra‐high‐temperature kinds of milk had lactose contents similar to those of raw milk, increasing their contents of saturated fatty acids, such as hexadecanoic acid and octadecanoic acid. However, the close composition outline in raw milk was not significantly changed by the ultra‐high‐temperature processes regarding its potential nutritive properties and consequent benefits for human health. These results may help to optimize milk processing.

## AUTHOR CONTRIBUTIONS

**Ge Bai:** Data curation (equal); formal analysis (equal); investigation (equal); writing – original draft (equal). **Long Cheng:** Data curation (equal); formal analysis (equal). **Liying Peng:** Data curation (equal); formal analysis (equal); investigation (equal); writing – original draft (equal). **Bin Wu:** Formal analysis (equal). **Yuguo Zhen:** Project administration (equal); writing – review and editing (equal). **Guixin Qing:** Writing – review and editing (equal). **Xuefeng Zhang:** Resources (equal). **Natnael D. Aschalew:** Writing – review and editing (equal). **Zhe Sun:** Conceptualization (equal). **Tao Wang:** Formal analysis (equal); supervision (equal); writing – review and editing (equal).

## FUNDING INFORMATION

This study was supported by the Jilin Province (China) Scientific and Technological Developing Scheme (20210202037NC, 20210508007RQ).

## CONFLICT OF INTEREST STATEMENT

We certify that no financial organization has a conflict of interest concerning the material discussed in this manuscript.

## Supporting information


Tables S1‐S2

## Data Availability

The data that support the findings of this study are available from the corresponding author upon reasonable request.
