# Supplementary material for: Effects of ultra‐high‐temperature processes on metabolite changes in milk
Source: Food Sci Nutr. 2023 Apr 6;11(6):3601–15. doi: 10.1002/fsn3.3350 (PMC10261746; doi:10.1002/fsn3.3350)
Supplement: Supplementary file 1 — Tables S1‐S2 [file FSN3-11-3601-s001.docx]

Supplementary Material

# Supplementary TABLES

**TABLE S1** Evaluation parameters of OPLS-DA models in the chloroform layers of milk samples.

| Treatment | R2X | R2Y^a^ | Q^2b^ |
| --- | --- | --- | --- |
| Raw milk vs Pasteurized milk | 0.932 | 0.902 | 0.87 |
| Pasteurized milk vs Semi-finished milk | 0.821 | 0.596 | 0.56 |
| Semi-finished milk vs UHT milk | 0.819 | 0.451 | 0.53 |
| UHT milk vs Finished milk | 0.767 | 0.768 | 0.607 |

^a^R2Y indicates the rate of model interpretation and Q^2^ indicates the model’s predictive ability.

^b^The R^2^ and Q^2^ more than 0.5 , the more stable and reliable the model is.

**TABLE S2** Evaluation parameters of OPLS-DA models in the water layers of milk samples.

| Treatment | R2X | R2Y^a^ | Q^2b^ |
| --- | --- | --- | --- |
| Raw milk vs Pasteurized milk | 0.748 | 0.713 | 0.535 |
| Pasteurized milk vs Semi-finished milk | 0.773 | 0.785 | 0.522 |
| Semi-finished milk vs UHT milk | 0.738 | 0.965 | 0.792 |
| UHT milk vs Finished milk | 0.696 | 0.816 | 0.711 |

^a^R2Y indicates the rate of model interpretation and Q^2^ indicates the model’s predictive ability.

^b^The R^2^ and Q^2^ more than 0.5 , the more stable and reliable the model is.
